# Impact of Stressful Life Events on Infant Birth Outcomes: Do Race/Ethnicity and Hypertension in Pregnancy Matter?

**DOI:** 10.1177/26884844251378359

**Published:** 2025-09-15

**Authors:** Forgive Avorgbedor, Thomas P. McCoy, Lori Hubbard, Amita Mittal, Stephanie Pickett

**Affiliations:** University of North Carolina at Greensboro School of Nursing, Greensboro, NC, USA.

**Keywords:** stressful life events, low birth weight, preterm birth, small for gestational age, PRAMS, ethnicity

## Abstract

**Background:**

Exposure to stressors impacts maternal and infant health. Growing evidence suggests stressful life events (SLEs) are associated with adverse infant birth outcomes. The objective of this study was to determine how SLEs affected infant outcomes (low birth weight [<2500 g], preterm birth, and small for gestational age), and how these effects were influenced by race/ethnicity and hypertensive disorders of pregnancy.

**Methods:**

The weighted prevalence of SLEs and adverse infant outcomes were investigated using data from the Pregnancy Risk Assessment Monitoring System (PRAMS) from 2009 to 2020. Adjusted risk ratios were estimated for the effects of SLEs, hypertensive disorders of pregnancy (including hypertension before pregnancy and pregnancy-induced hypertension), and race/ethnicity on infant outcomes using multivariable log-binomial modeling.

**Results:**

The dataset included 452,031 women between the ages of 18 and 45. During the year before giving birth, the incidence of self-reported SLEs was between 66% and 72%. Black mothers had more SLEs, significantly increased risk of preterm birth (<37 weeks) and low birth weight.

**Conclusion:**

Adverse infant outcomes were more likely among Black mothers participating in PRAMS, perhaps because of a higher number of self-reported SLEs.

## Background

Growing evidence suggests that exposure to stressors—whether emotional (*e.g.,* someone very close died), partner-associated (*e.g.,* husband/partner said he did not want pregnancy), or traumatic stressors (*e.g.,* someone very close had a problem with drinking or drugs)^1^—increases the risk of adverse pregnancy outcomes. These adverse outcomes include preterm birth and infants with low birth weight or small for gestational age (SGA).^2–4^ Self-report data from the Pregnancy Risk Assessment Monitoring System (PRAMS) from 2009 to 2011 indicate that approximately 70% of pregnant women in the United States experience at least one stressful life event (SLE) during the year before giving birth.^5^ Lifetime exposure to psychosocial stress also is associated with a higher likelihood of being diagnosed with hypertensive disorders of pregnancy (HDP), including hypertension (HTN) before pregnancy and pregnancy-induced hypertension (PIH).^6^ Preconception life stressors, such as financial ones, are associated with preterm birth.^7^ Black women report experiencing SLEs at a higher rate compared to White women,^1^ and the prevalence of adverse pregnancy outcomes is higher among Black women than White women.^8–11^

Systematic reviews have reported associations between stress and adverse infant outcomes, such as low birth weight.^12,13^ Furthermore, higher cortisol levels are associated with adverse birth outcomes, especially in low-income and minority pregnant women, supporting the concept that stress is linked to adverse birth outcomes.^14–16^ Compared to White women, Black women in the United States continue to have a higher rate of adverse birth outcomes, including low birth weight and SGA infants.^8,9^

Multiple factors, including psychosocial stress, lifetime stressors, and preconception SLEs, contribute to HDP and adverse infant outcomes.^6,7,11^ Previous studies using PRAMS data have reported on SLEs among women a year before childbirth across multiple states,^1,5^ and on SLEs, HDP, and preterm birth in one state with a relatively small sample.^7^ These data were collected from 2000 to 2010, 2009 to 2011, and 2012 to 2014. More information is needed to determine the impact of SLEs on adverse infant outcomes, including low birth weight, SGA, and preterm birth in relation to HDP and race/ethnicity. Therefore, the objective of this study was to determine the effects of SLEs on infant outcomes (low birth weight, preterm birth, and SGA) and understand these effects by race/ethnicity and HDP over 11 years of PRAMS data. This study was approved by the Centers for Disease Control and Prevention (CDC) in accordance with the data usage agreement for PRAMS. All women provided consent to participate in PRAMS.

## Methods

### Participants

PRAMS is a United States surveillance program conducted by state health departments and the CDC annually. Random samples of mothers who had a live birth are invited to participate in PRAMS data collection, including a follow-up phone call. If the response rate among those invited mothers meets the threshold set by the CDC for a given year, the CDC will include those state data in their United States-wide data collection. From 2009 to 2020, PRAMS was conducted in 38–50 sites, but the release of data by site varies by year (range 27–43). Data collection includes self-reported behaviors at prenatal, perinatal, and postnatal stages. More details about PRAMS can be found in the report by Shulman and colleagues (2018).^17^ PRAMS data are publicly available through the CDC. Ethical approval was not necessary for this study.

### Measures

PRAMS asks questions about stressful events in the 12 months before the current infant’s birth. Examples of items included: “I got separated or divorced from husband or partner” and “I lost my job even though I wanted to continue working.” The Yes responses to the SLE questions were summed, and the prevalence of at least one SLE in the past 12 months was captured.^1^ A one-factor categorical confirmatory factor analysis model estimated using weighted least squares estimation revealed good construct validity (root mean square error of approximation = 0.064), with factor loadings of 0.4 or higher except for the item: (0.205). Internal consistency was good (Kuder–Richardson formula −20 = 0.66).

### Race/Ethnicity

Because some racial/ethnic groups had smaller unweighted counts, race/ethnicity was categorized as Black (non-Hispanic), Hispanic, White (non-Hispanic), or all other (subsequently denoted other).

#### Hypertensive disorders of pregnancy

##### Hypertension before pregnancy

 HTN before pregnancy was captured with a single Yes/No self-reported question: “During the *3 months before* you got pregnant with your *new* baby, did you have any of the following health conditions? High blood pressure or hypertension.”

##### Pregnancy-induced hypertension

PIH was measured with a single Yes/No question for the seven states that opted to include it: “During *your most recent* pregnancy, did you have any of the following health conditions? High blood pressure (that started during this pregnancy), preeclampsia, or eclampsia.”

##### Small for gestational age

Infants with a birth weight below the 10th percentile for the same weeks of pregnancy (gestational age) were considered SGA.^18^

##### Preterm birth

Infants born before 37 weeks of pregnancy were considered preterm.

##### Low birth weight

Infants who weighed <2500 g at birth were considered to have low birth weight.

### Conceptual model

**FIG. 1.**
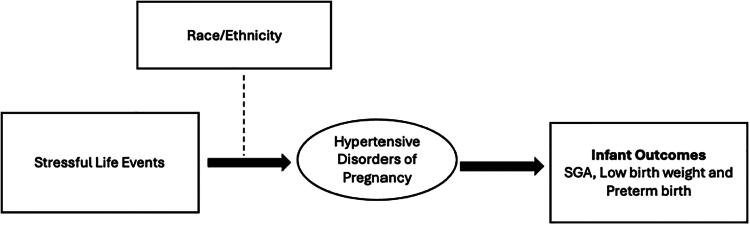
Conceptual model underlying the study. SGA, small for gestational age.

Our conceptual model considered the influence of SLEs on adverse infant outcomes, especially HDP (see Fig. 1). HDP is positioned as a key biological factor linking psychosocial stress to adverse perinatal outcomes. We posit that the effects of SLEs and HDP may differ across racial and ethnic groups due to structural factors, differential exposure to stressors, and variable physiological responses.

### Statistical methods

Weighted percentages for states and overall, by year, were estimated to investigate trends over time. Multivariable modeling of dichotomous infant outcomes was performed using log-binomial modeling^19^ to estimate adjusted relative risk ratios and their 95% confidence intervals (CI). Of interest were the interactions between race/ethnicity groups, HTN before pregnancy, PIH, and year, when applicable. Subpopulation domain analysis^20^ was performed for study inclusion criteria of those with a single parity and maternal age between 18 and 45 (inclusive) and separately for the seven states that included questions for PIH. All analyses were weighted and adjusted for the PRAMS design (*e.g.,* oversampling of low birth weight infants; Shulman et al.^17^) Analyses were performed in SAS v9.4 (SAS Institute, Cary, NC) and STATA v18.0 (STATA Corp., College Station, TX). A two-sided *p*-value <0.05 was considered statistically significant.

## Results

Participants included 452,031 women between the ages of 18 and 45 sampled from 2009 to 2020. For modeling of infant outcomes after subpopulation analyses, the unweighted sample size ranged from 291,162 to 292,135 (weighted *N* range, 15,828,115–15,850,391 women). The proportion of participants with self-reported SLEs ranged from 66% to 72%.

**FIG. 2.**
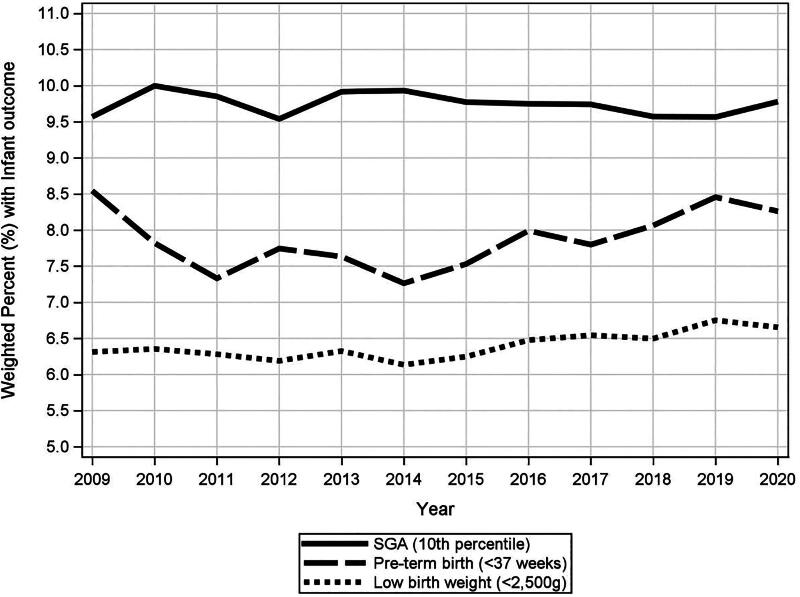
Trends over time in infant outcomes: small for gestational age (SGA), preterm birth, and low birth weight.

We previously reported trends of SLEs from 2009 to 2020, SLEs by race/ethnicity, and HDP by race/ethnicity.^21^ Figure 2 shows longitudinal trends in infant outcomes. While rates of SGA and low birth weight among infants had similar trends, rates of preterm birth were more variable. Rates of SGA ranged from 9.6% in 2009 to 9.8% in 2020, low birth weight from 6.3% in 2009 to 6.7% in 2020, and preterm births ranged from 8.5% in 2009 to 8.3% in 2020. The lowest rate for preterm births was 7.3% in 2014. There were no significant interactions between SLEs and year for unadjusted models (all *p* > 0.05).

**FIG. 3.**
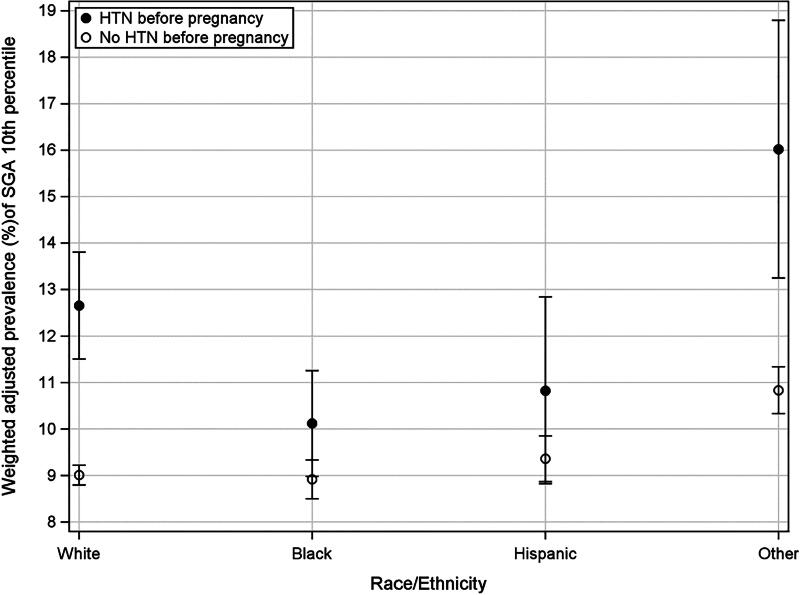
Adjusted risk of small for gestational age (SGA, 10th percentile) by race/ethnicity × hypertension before pregnancy (error bars = 95% CI). CI, confidence intervals.

SLEs were associated with a significantly increased risk of SGA (1.012; 95% CI: 1.003–1.021). There were no significant three-way interaction effects of race/ethnicity, HTN before pregnancy, and year. After testing all two-way interactions, only race/ethnicity × HTN before pregnancy differences were significant (*F*[3, 327,736] = 3.52, *p* = 0.014). Interactions between race/ethnicity × year (*F*[33, 327,706] = 0.86, *p* = 0.694) and HTN before pregnancy × year (*F*[11, 327,728] = 1.20, *p* = 0.282) were not significant. A final model with just race/ethnicity × HTN before pregnancy pairwise interactions (*F*[3, 327,736] = 3.76, *p* = 0.010) is presented in Table 1. Lower age, lower education, not being married, income below the poverty level, no insurance, and smoking during the third trimester all significantly increased the risk of SGA (all *p* < 0.05). For effects of race/ethnicity and HTN before pregnancy, adjusted risk (margins) were estimated by combining interaction categories (Fig. 3). Women with HTN before pregnancy had a higher risk of delivering SGA infants across race/ethnicity groups, though the extent depended on their specific race/ethnicity. Non-Hispanic White women (12.7% with HTN vs. 9.0% with no HTN) and women of other races/ethnicities (16.0% with HTN vs. 10.8% with no HTN) had the greatest differences in predicted risk of delivering SGA infants. For the seven states asking about PIH, adjusted effects of PIH on SGA 10th percentile were not race-specific (interaction *F*[3,66,726] = 0.63, *p* = 0.595). Women with HDP had a 50.0% higher risk of having SGA, adjusting for other model characteristics (ARR = 1.500, 95% CI = [1.368, 1.644], *p* < 0.001).

**Table 1.** Final Multivariable Log-Binomial Model of Risk for Infants Who Were Small for Gestational Age (10th Percentile)

Independent variable	ARR	95% CI for ARR	*p*
Maternal age (years)	0.992	0.989, 0.996	<0.001
Education >12 years	0.925	0.886, 0.865	<0.001
Married versus otherwise	0.882	0.844, 0.922	<0.001
Body mass index (overweight/obese vs. normal)	0.735	0.709, 0.761	<0.001
Below poverty income level	1.104	1.052, 1.159	<0.001
Insurance			
Private	0.951	0.905, 0.994	0.041
Not private	0.948	0.905, 0.994	0.026
None (RC)	—	—	—
Maternal diabetes (DM) prepregnancy	0.758	0.675, 0.851	<0.001
Gestational DM during pregnancy	0.849	0.799, 0.902	<0.001
Used alcohol prepregnancy	0.986	0.951, 1.022	0.427
Any smoking during 3rd trimester	1.947	1.846, 2.053	<0.001
Stressful life events scale, sum	1.012	1.003, 1.021	0.011
Race/Ethnicity			
Black	0.990	0.938, 1.045	0.725
Hispanic	1.040	0.980, 1.103	0.197
Other	1.203	1.142, 1.267	<0.001
White (RC)	—	—	—
HTN before pregnancy	1.405	1.279, 1.543	<0.001
Race/Ethnicity × HTN before pregnancy			
Black with HTN before pregnancy	0.807	0.696, 0.937	0.005
Hispanic with HTN before pregnancy	0.823	0.666, 1.016	0.070
Other with HTN before pregnancy	1.052	0.865, 1.279	0.611

Unweighted *n* = 291,162; weighted *N* = 15,828,115. Also adjusted for year; year effects not shown.

ARR, adjusted relative risk; CI, confidence intervals; RC, reference category.

For preterm birth, SLEs were associated with a significantly increased risk of preterm birth (adjusted relative risk 1.019 [95% CI: 1.010–1.029]). There were no significant three-way interaction effects of race/ethnicity, HTN before pregnancy, and year. Testing all two-way interactions of these variables revealed no significant pairwise interaction effects: race/ethnicity × HTN before pregnancy (*F*[3, 328,286] = 0.11, *p* = 0.955), race/ethnicity × year (*F*[33, 328,256] = 1.09, *p* = 0.328), and HTN before pregnancy × year (*F*[11, 328,278] = 1.52, *p* = 0.116). A final model with just the main effects is presented in Table 2. Non-Hispanic Black women had a 41.3% higher risk of preterm birth relative to non-Hispanic White women, and Hispanic women had a 6.6% increased risk of preterm birth versus White women. Other factors associated with increased risk of preterm birth were greater maternal age, less than 12 years of education, not being married, overweight/obesity, income below the poverty level, no insurance versus public insurance, maternal prepregnancy diabetes mellitus, gestational diabetes mellitus, and smoking during the third trimester (all *p* < 0.05). Women with PIH had 159% higher risk of having a preterm birth, after controlling for other model characteristics (adjusted relative risk 2.592, 95% CI = [2.389, 2.811]; *p* < 0.001). However, PIH effects on preterm birth were not race-specific (interaction *F*[3,66,726] = 2.03, *p* = 0.108).

**Table 2.** Final Multivariable Log-Binomial Model of Preterm Birth (<37 Weeks Gestation)

Independent variable	ARR	95% CI for ARR	*p*
Maternal age (years)	1.017	1.013, 1.020	<0.001
Education >12 years	0.875	0.839, 0.912	<0.001
Married versus otherwise	0.843	0.807, 0.881	<0.001
BMI overweight/obese versus normal	1.096	1.059, 1.135	<0.001
Below poverty income level	1.075	1.024, 1.129	0.003
Insurance			
Private	1.038	0.991, 1.088	0.115
Not private	1.082	1.033, 1.134	0.001
None (RC)	—	—	—
Maternal diabetes (DM) prepregnancy	1.385	1.270, 1.510	<0.001
Gestational DM during pregnancy	1.288	1.221, 1.359	<0.001
Used alcohol prepregnancy	0.900	0.868, 0.933	<0.001
Any smoking during 3rd trimester	1.242	1.169, 1.320	<0.001
Stressful life events scale sum	1.019	1.010, 1.029	<0.001
Race/Ethnicity			
Black	1.413	1.349, 1.479	<0.001
Hispanic	1.066	1.008, 1.128	0.026
Other	1.050	0.994, 1.109	0.082
White (RC)	—	—	—
HTN before pregnancy	1.605	1.509, 1.707	<0.001

Unweighted *n* = 291,712; weighted *N* = 15,828,768. Additionally adjusting for year; year effects not shown.

**FIG. 4.**
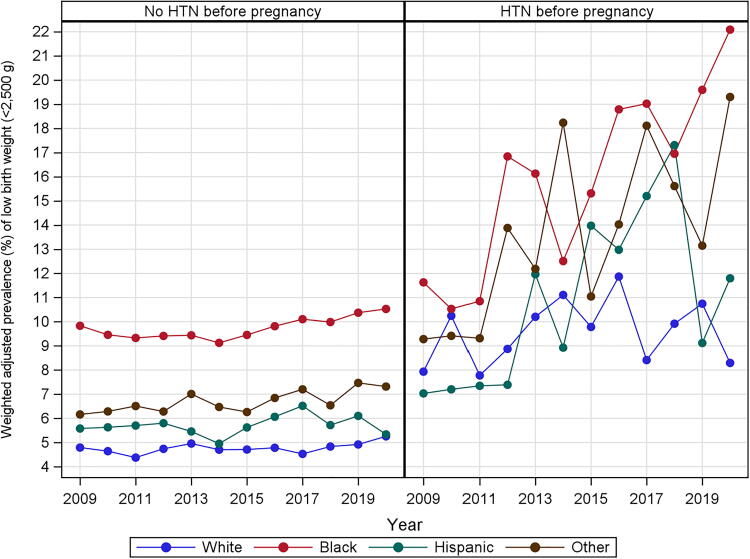
Adjusted risk of low birth weight (<2500 g) for race/ethnicity and HTN before pregnancy by year. HTN, hypertension.

SLEs were associated with a significantly increased risk of low birth weight (adjusted relative risk 1.017 [95% CI: 1.010–1.025]). There were no significant three-way interaction effects of race/ethnicity, HTN before pregnancy, or year (*F*[33, 328,679] = 1.13, *p* = 0.282). Testing all two-way interactions of these variables revealed that race/ethnicity × HTN before pregnancy differences (*F*[3, 328,709] = 5.11, *p* = 0.002) and HTN before pregnancy × year (*F*[11, 328,701] = 3.35, *p* < 0.001) differed significantly, while race/ethnicity × year (*F*[33, 328,679] = 0.87, *p* = 0.687) did not. A final model with pairwise interactions for race/ethnicity × HTN before pregnancy (*F*[3, 328,709] = 5.11, *p* = 0.002) and HTN before pregnancy × year (*F*[11, 328,701] = 3.52, *p* < 0.001) is presented in Table 3. Adjusted risks were estimated by the interaction category combinations and are plotted in Figure 4.

**Table 3.** Final Multivariable Log-Binomial Model of Low Birth Weight (<2500 g)

Independent variable	ARR	95% CI for ARR	*p*
Maternal age (years)	1.011	1.008, 1.014	<0.001
Education >12 years	0.884	0.852, 0.916	<0.001
Married versus otherwise	0.781	0.753, 0.811	<0.001
BMI overweight/obese versus normal	0.887	0.861, 0.914	<0.001
Below poverty income level	1.079	1.037, 1.123	<0.001
Insurance			
Private	1.012	0.972, 1.055	0.552
Not private	1.043	1.003, 1.085	0.034
None (RC)	—	—	—
Maternal diabetes (DM) prepregnancy	0.932	0.853, 1.018	0.117
Gestational DM during pregnancy	1.059	1.011, 1.110	0.016
Used alcohol prepregnancy	0.914	0.887, 0.943	<0.001
Any smoking during 3rd trimester	1.683	1.601, 1.769	<0.001
Stressful life events scale sum	1.017	1.010, 1.025	<0.001
Race/Ethnicity			
Black	2.040	1.957, 2.126	<0.001
Hispanic	1.195	1.143, 1.250	<0.001
Other	1.402	1.337, 1.470	<0.001
White (RC)			
HTN before pregnancy	1.489	1.248, 1.776	<0.001
Race/Ethnicity × HTN before pregnancy			
Black with HTN before pregnancy	0.817	0.732, 0.914	<0.001
Hispanic with HTN before pregnancy	0.940	0.807, 1.095	0.427
Other with chronic HTN	1.018	0.863, 1.202	0.829
Year × HTN before pregnancy			
2010 with HTN before pregnancy	1.052	0.837, 1.323	0.661
2011 with HTN before pregnancy	1.024	0.815, 1.286	0.840
2012 with HTN before pregnancy	1.321	1.045, 1.671	0.020
2013 with HTN before pregnancy	1.366	1.082, 1.725	0.009
2014 with HTN before pregnancy	1.413	1.105, 1.807	0.006
2015 with HTN before pregnancy	1.401	1.126, 1.744	0.002
2016 with HTN before pregnancy	1.579	1.258, 1.883	<0.001
2017 with HTN before pregnancy	1.478	1.162, 1.879	0.001
2018 with HTN before pregnancy	1.469	1.173, 1.840	0.001
2019 with HTN before pregnancy	1.441	1.158, 1.792	0.001
2020 with HTN before pregnancy	1.465	1.173, 1.829	0.001

Unweighted *n* = 292,135; weighted *N* = 15,850,391. Additionally adjusting for year; year main effects not shown.

There were increasing trends for low birth weight over time, and Black women had the highest estimated risk of low birth weight. Other maternal factors associated with increased risk of low birth weight were greater age, less than 12 years of education, not being married, income below the poverty level, no insurance versus public insurance, gestational diabetes, and smoking during the third trimester (all *p* < 0.05). For the states capturing PIH, a significant race × PIH interaction was found (*F*[3,66,825] = 7.68, *p* < 0.001). The adjusted predicted risk of low birth weight by race was as follows: 11.4% with PIH versus 3.7% no PIH for White, 19.7% PIH versus 8.9% no PIH for Black, 15.2% PIH versus 4.5% no PIH for Hispanic, and 20.6% PIH versus 6.3% no PIH for other race.

## Discussion

The present study explored SLEs and their impact on infant outcomes, and these effects were further assessed based on race/ethnicity, HTN before pregnancy, and PIH. Using national PRAMS data from 2009 to 2020, rates of self-reported SLEs are consistently above 66%. SLEs were associated with SGA, preterm birth, and low birth weight, with significant interactions for SGA and low birth weight. Additionally, women with PIH were more likely to have preterm infants, and race/ethnicity was associated with having low birth weight infants. Although previous studies have shown that SLEs are associated with adverse pregnancy outcomes,^1,5^ this study clearly demonstrates associations between SLEs and SGA, preterm birth, low birth weight, and hypertension.

A previous study that reported on PRAMS data from 2000 to 2010 indicated a slight but significant decrease in SLEs, with 70.2% of participants reporting at least one SLE.^1^ A study that used PRAMS data from 2009 to 2011 reported that 70% of the respondents had at least one SLE a year before delivery.^5^ In a study using results from the PRAMS database for 1990–1995, 64% of women reported greater than or equal to one SLE a year before delivery.^22^ We found that SLEs reported from 2009 to 2020 were between 66% and 73%. Overall, these results indicate that the prevalence of SLEs among reproductive women has been consistently high over 30 years.

The associations observed in the current study between SLEs and SGA, preterm birth, and low birth weight were consistent with previous studies indicating that SLEs are associated with SGA,^23^ preterm birth,^5,24^ and low birth weight.^25^ Findings in the present study also revealed an interaction between race/ethnicity and HTN before pregnancy, which was associated with SGA and low birth weight. Non-Hispanic White women and women of other race/ethnicity with HTN before pregnancy are at a higher risk of delivering SGA infants compared to their counterparts without HTN. In addition, non-Hispanic women had a higher risk for SGA compared to non-Hispanic Black and Hispanic women. Previous studies had reported lower SGA birth rates among non-Hispanic Whites compared to non-Hispanic Blacks and Hispanics.^26,27^ This finding could be explained by higher prepregnancy risk for SGA among non-Hispanic Black women, often driven by structural factors and social determinants of health.^26,27^ Additionally, differences in diagnosis and management of HTN before pregnancy may contribute to non-Hispanic White women receiving earlier detection and more intensive monitoring, leading to increased identification of SGA. In contrast, underdiagnosis or undertreatment among non-Hispanic Black women could mask effects of HTN before pregnancy.^28^

For non-Hispanic Black women, the risk for delivering SGA infants is independent of HTN before pregnancy, and this finding is consistent with a previous study.^28^ On the other hand, non-Hispanic Black women with HTN before pregnancy had a higher risk of preterm birth compared to women without hypertension. Women with HTN before pregnancy are more likely to be Black and to deliver preterm and SGA infants.^29,30^ These findings add to the existing evidence on addressing preexisting chronic diseases among reproductive-age women.

We found that PIH increases the risk of preterm birth independent of race/ethnicity, consistent with previous studies of PIH and adverse infant outcomes.^31^ However, the risk for low birth weight infants was consistently higher across all races/ethnicities among women with PIH. These findings highlight the need for effective management and innovative intervention strategies for women at high risk for pregnancy complications.

Although the absolute risk increase per unit change in SLEs appears small, even modest relative risks can translate into a considerable public health burden when exposure to SLEs is highly prevalent. SLEs likely act synergistically with other risk factors, such as structural factors, inadequate access to care, and HDP. Our findings are consistent with the literature, which demonstrates that chronic stressors exerts cumulative biological effects over time. Thus, our results support the importance of addressing SLEs as part of a broader strategy to reduce disparities in adverse birth outcomes.

### Limitations

This study used data from states that submitted their data to the CDC. Participating statements are only submitted to the CDC if they meet the set threshold of a particular year. Thus, bias could result from states where data were below the threshold and thus unreported. Nonetheless, the complex sampling design in PRAMS data makes generalizability of its results more likely. Since data are cross-sectional, we cannot ascertain causality, and factors may also be subject to recall bias.

Our analyses of PIH were limited to data from seven states that regularly reported on PIH during 2019 and 2020. Furthermore, HDP rates were self-reported. However, these were cross-checked with birth certificate records, a reliable source for HDP and pregnancy maternal health information.^32–34^

At the policy level, efforts to reduce social and structural stressors during pregnancy, such as strengthening paid family leave, could help mitigate risks. Integrating perinatal mental health screening and support into routine prenatal care, and enhanced monitoring of women at risk for HDP, may improve early detection and management. Finally, programs that foster social support, such as group prenatal care and community health worker initiatives, could mitigate the impact of stress during pregnancy.

## Conclusion

Negative infant outcomes were more likely among Black mothers participating in PRAMS, perhaps because of a higher number of self-reported SLEs. Future interventions might help improve prenatal mental health for expectant mothers and their subsequent pregnancy and birth experiences. Routine screening for SLEs among reproductive-age women and improving access to early prenatal care could reveal potential issues and proactively engage further resources.

## Data Availability

PRAMS data analyzed during the current study are not publicly available due to restrictions from the Centers for Disease Control and Prevention. The PRAMS data can be requested from the Centers for Disease Control and Prevention at https://www.cdc.gov/prams/index.html.

## Abbreviations Used

AAR: adjusted relative risk
CDC: Centers for Disease Control and Prevention
CI: confidence intervals
HDP: hypertensive disorders of pregnaancy
HTN: hypertension
PIH: pregnancy-induced hypertension
PRAMS: Pregnancy Risk Assessment Monitoring System
SGA: small for gestational age
SLEs: Stressfull life events
